# Distinct changes in global brain synchronization in different motor subtypes of Parkinson’s disease

**DOI:** 10.3389/fnins.2023.1170225

**Published:** 2023-10-18

**Authors:** Chendie Song, Qin Shen, Changlian Tan, Junli Li, Fan Zhou, Tianyu Wang, Lin Zhang, Min Wang, Yujing Liu, Jiaying Yuan, Sainan Cai, Haiyan Liao

**Affiliations:** ^1^Department of Radiology, The Second Xiangya Hospital, Central South University, Changsha, China; ^2^Department of Radiology, Affiliated Hangzhou First People’s Hospital, Zhejiang University School of Medicine, Hangzhou, China

**Keywords:** Parkinson’s disease, degree centrality, brain network, global brain synchronization, resting-state functional MRI

## Abstract

This study investigated alterations in degree centrality (DC) in different motor subtypes of Parkinson’s disease (PD) and analyzed its clinical significance during disease occurrence. A total of 146 subjects were recruited in the study, including 90 patients with PD [51 and 39 with tremor dominant (TD) and akinetic-rigid dominant (ARD) disease, respectively] and 56 healthy controls (HCs). The resting-state functional magnetic resonance imaging data of all the subjects were obtained by 3.0 T magnetic resonance scans. The DC values, an indicator of whole brain synchronization, were calculated and compared among the TD, ARD, and HC groups. Disparities in DC values among the three groups were evaluated by analysis of variance and *post hoc* two-sample *t*-tests. Correlation between brain regions with DC differences and clinical variables were performed using partial correlation analysis after controlling for age, gender, and disease duration. Compared to the HCs, both TD and ARD groups demonstrated increased DC values bilaterally in the cerebellum; DC values were decreased in the left putamen and paracentral lobule in the TD group and in the left anterior cingulate gyrus and right supplementary motor area in the ARD group. Compared to the ARD group, the TD group showed decreased DC values in bilateral cerebellar hemispheres and increased DC values in the left anterior cingulate gyrus and right supplementary motor area. The DC of the whole brain showed inconsistencies and shared neural bases among patients with the two subtypes of PD. The differences between brain regions with abnormal DC values may be closely related to different clinical presentations of the two motor subtypes. Our findings provide new insights into the clinical heterogeneity of PD with respect to different motor subtypes.

## 1. Introduction

Parkinson’s disease (PD) is a progressive neurodegenerative disease of older adults, and is the second most common such condition after Alzheimer’s disease. It presents with typical motor symptoms including resting-tremor, rigidity, bradykinesia, and postural instability and several other non-motor symptoms such as anxiety, depression, and sleep disturbance, among others (Chaudhuri et al., 2006; Lees et al., 2009; Kalia and Lang, 2015). Patients with PD may be divided into two major subtypes according to the predominant motor symptoms, namely, tremor-dominant (TD) and akinetic-rigid dominant (ARD); mixed subtypes are also seen (Marras et al., 2002; Xiang et al., 2017). Clinical evidence suggests that patients with different motor subtypes of PD have distinct clinical courses and prognoses (Rajput et al., 2009). Compared to the TD subtype, the ARD subtype tends to be associated with more rapid clinical progression and a higher risk of dementia and other mental diseases such as depression; this suggests disparities in neural bases among the different subtypes (Vu et al., 2012; van der Heeden et al., 2016; Mohl et al., 2017). However, the underlying neuro-pathological mechanisms in different motor subtypes of PD remain unclear.

Previous neuroimaging studies have shown different patterns of brain alterations between the TD and ARD subtypes of PD (Eggers et al., 2011; Zhang et al., 2015; Karunanayaka et al., 2016; Zhang et al., 2016; Piccinin et al., 2017). Several studies using positron emission tomography and single photon emission computed tomography have shown that patient expression of a previously validated PD related metabolic covariance pattern correlates with clinical scores for akinesia and rigidity, but not tremor; this suggests the possible role of different neurobiological substrates in Parkinsonian tremors, unlike those of akinesia and rigidity (Eidelberg et al., 1995; Mure et al., 2011). A voxel-based morphological study showed that patients with TD exhibit reduced grey matter volume in the cerebellum (Benninger et al., 2009); this indicates that the cerebellum may play an important role in patients with PD.

Blood oxygenation level-dependent resting-state functional MRI (rs-fMRI) has been used widely in recent years to examine brain functions in patients with PD at the system level (Fox and Raichle, 2007; van Eimeren et al., 2009). The study of spontaneous brain activity is currently an area of particular interest for functional neuroimaging studies on different motor subtypes of PD; evaluation is mainly based on regional homogeneity (Yang et al., 2013; Jiang et al., 2016; Hu et al., 2019), the amplitude of low-frequency fluctuations, and functional connectivity (FC) (Xiang et al., 2016; Pang et al., 2021). However, the information provided by the rs-fMRI markers described above is limited. For example, regional homogeneity and the amplitude of low-frequency fluctuations, as observed using functional separation methods, only reflect local functional alterations, and information on functional connections may be excluded at the whole-brain level. In contrast, FC analysis requires prior assumption, which may lead to selection bias. As we all know, based on the functional connection analysis of seed points or regions of interest, researchers often preset a target brain region as the starting point of research based on the patient’s clinical symptoms and previous research results, and observe the functional connection changes between other brain regions in the PD patient’s brain and the target brain region, which may make the research results limited by the brain region, and secondly, the research results will be largely related to the preset brain region, resulting in the selection bias of the investigator. Degree centrality (DC), a reliable rs-fMRI parameter (Zuo et al., 2013) used for exploring whole-brain neural network abnormalities without assumptions, has recently gained more attention. It can reflect the FC strength in the whole brain at the voxel level, rather than being limited to changes in a single brain region, to a certain extent, making the changes in the brain more holistic. Previous studies have shown that changes in DC are related to depression and motor dysfunction in patients with PD (Wang et al., 2018; Zhang et al., 2019). However, alterations in the global synchronization of different motor subtypes of PD have not been reported.

As studies exploring motor subtypes of PD using DC are lacking, the present study evaluated and compared whole-brain DC alterations between the TD and ARD subtypes. This was performed to explore the potential neural mechanisms responsible for the clinical heterogeneity of different motor subtypes of PD.

## 2. Materials and methods

### 2.1. Participants

The present study was approved by the local hospital medical ethics committee. Particular emphasis was placed on the acquisition of informed consent from all participants.

All patients with PD were enrolled from the outpatient department of the Second Xiangya Hospital of the Central South University between February 2018 and March 2021. Each patient was diagnosed with idiopathic PD by two experienced neurologists based on the Movement Disorder Society criteria for PD (Postuma et al., 2015). The inclusion criteria were as follows: (1) right handedness, (2) Hoehn and Yahr stage ≤3.0, (3) no obvious cognitive impairment (Li et al., 2016), (4) clinically in the off state, and (5) no history of severe systemic or other neurological or psychiatric diseases. In addition, patients with contraindications to MRI or those having a history of chronic drug or alcohol abuse were excluded. The patients were then asked to stop taking medicines prescribed for PD from 12 h before the MRI scan. All participants underwent extensive neuropsychological evaluation before the scan. Data pertaining to demographic characteristics of the subjects, including age, sex, and disease duration were collected. Patients were assessed using clinical scales, including the Unified Parkinson’s Disease Rating Scale (UPDRS) scale (including four parts, non-motor symptoms of daily life, motor symptoms of daily life, functional tests of exercise and motor complications), Mini-mental State Examination (MMSE) scale (assessing the patient’s cognitive function), Hoehn & Yahr stage (assessing the severity of the patient), and the 17-item Hamilton Depression (HAMD-17) scale (assessment of depressive states) when they were in the off state. The daily levodopa equivalent dose was also calculated in the patients who received medications. Based on the method proposed by Kang et al., patients with PD were divided into different subtypes based on the individual ratio of the UPDRS III tremor score to the mean UPDRS akinetic/rigid score (Kang et al., 2005). Furthermore, a ratio was individually calculated between the mean UPDRS III tremor score (the mean of the component sub-items of items 20 and 21) and the mean UPDRS akinetic/rigid score (the mean of the component sub-items of items 22–27 and 31). Patients with ratios of >1.0, <0.8, and 0.8–1 were categorized into TD, ARD, and mixed subtypes, respectively. Age- and sex-matched HC subjects, excluding those with a history of severe psychiatric or organic neurological disease, depressive tendencies and cognitive impairment, were recruited from the local community; these subjects were also assessed through unstructured clinical interviews.

Overall, 100 patients with PD were included in our study along with 56 HC subjects. The patients were divided into three groups, with 51, 39, and 10 patients having the TD, ARD, and mixed subtypes, respectively. As the sample size of the mixed subtype was relatively small, the data of patients from this subtype were not included for subsequent analyses.

### 2.2. MR data acquisition and preprocessing

Whole-brain MRI data of all subjects were acquired using a 3.0 T MRI scanner (MAGNETOM Skyra; Siemens Healthineers, Germany) with a 16-channel phased array coil at the Department of Radiology of the Second Xiangya Hospital, Central South University. T1-weighted three-dimensional magnetization-prepared rapid acquisition gradient echo images and whole-brain functional scans were obtained. The scan sequences and parameters were as follows: T1-weighted three-dimensional magnetization-prepared rapid acquisition gradient echo images: 176 axial slices with no-spacing scan, slice thickness = 1.0 mm, repetition time = 1,900 ms, echo time = 2.01 ms, flip angle = 9°, and field of view = 256 × 256 mm^2^; whole-brain functional images: 39 axial slices with no-spacing scan, slice thickness = 3.5 mm, repetition time = 2,500 ms, echo time = 25 ms, flip angle = 90°, field of view = 240 × 240 mm^2^, matrix = 64 × 64, and voxel size = 3.8 × 3.8 × 3.5 mm^3^; 200 volumes. During rs-fMRI sequence acquisition, all participants were asked to keep their eyes closed, not to fall asleep, and not to focus their thoughts on anything. In order to reduce the discomfort of the subjects during the examination and ensure the quality of the scanned images, each subject used earplugs to reduce noise and a foam pad was placed on the head to limit head movement.

The rs-fMRI data were preprocessed on the MATLAB R2013b platform (The Math Works Inc., Natick, MA, United States) using Data Processing Assistant for rs-fMRI Data Analysis Toolkit (REST plus) software version 1.21 (Jia et al., 2019), as previously described (Liao et al., 2020; Wang et al., 2020). In summary, preprocessing involved the following eight steps: first, data were converted from digital imaging and communication in medicine to Neuroimaging Informatics Technology Initiative format for easy recognition and reading by post-processing software; second, the initial 10 volumes of all functional time series were removed to eliminate instability of the collected image caused by several factors such as machine heating, noise, or absence of a stable state at the beginning of the scan (due to head movement and environment, among others). Third, slice timing was performed; as the acquisition time of each slice of the image differs (sequential or interleaved scan), subsequent analysis considers all slices to be acquired at the same time. Fourth, realignment was performed and head movement due to involuntary head movement and machine vibrations were evaluated, as movements cause spatial displacement of the image and signal distortion (blur) (images with the subject’s head moving more than 0.5 mm or with angular rotation exceeding 0.5° in any direction were excluded). Fifth, spatial normalization was performed, as differences in individual head size and shape make it impossible to compare and count the same voxels among individuals. Sixth, the linear trend generated was removed to avoid signal drift that occurred during machine scans. Seventh, regression of covariates (including six head motion parameters, white matter, and cerebrospinal fluid signals) (Yan et al., 2013); and eighth, filtering was performed with a band pass of 0.01–0.08 Hz to eliminate certain confounding signals (such as signals generated by certain physiological activities).

### 2.3. DC calculation

DC plots of each subject were obtained using the RESTPLUS toolkit (version 1.21), following the classic method. As mentioned earlier, DC is a count of the total number of connections at the voxel level (Buckner et al., 2009; Zuo et al., 2012). We first used the preset prior probabilistic gray matter template in Statistical Parametric Mapping 8 software^1^ as a template for calculating the Pearson correlation coefficient between each voxel in all subjects and all remaining whole brain voxel time processes within the template. Each subject was then conferred a whole-brain Pearson correlation coefficient matrix. The DC was subsequently calculated with reference to the threshold of correlation of *r* > 0.25 based on a previous study. Finally, Fisher’s *r*-to-*z* transformation was employed to normalize the individual correlation matrix and a Gaussian smoothing kernel with a full width half maximum of 6 mm was used for spatial smoothing.

### 2.4. Statistical analysis

Statistical analysis of demographic information and correlation were performed by Statistical Package for the Social Sciences (SPSS) 25.0 software. We tested data for normality with the Kolmogorov–Smirnov Test; Levene’s Test was used to evaluate the homogeneity of variance. The variables of age in ARD group and education level in TD group fitted the normal distribution and showed homogeneity of variance, but the other clinical variables did not (*p* < 0.05). Thus, differences in age and education level across the TD, ARD, and HC groups were compared by Kruskal–Wallis (K–W) H test, differences of the variables of non-normal distribution between groups, including disease duration, UPDRS score, and UPDRS-III score, HY stage, MMSE score and HAMD-17 score, were compared with the Mann–Whitney U test. In addition, the Pearson chi-square test tested the gender distribution between groups.

One-way analysis of variance was performed using the RESTPLUS toolkit to compare differences in DC values among HC and the TD and ARD groups; age, gender, and education were considered as covariates (the significance threshold was considered at *p* < 0.001 at the voxel level with binding voxel sizes >13; this corresponded to *p* < 0.05 for a two-tailed test, as corrected by the AlphaSim program). Subsequently, a *post hoc* two-sample *t*-test was used to compare differences between each pair of the three groups (TD vs. HC, ARD vs. HC, and TD vs. ARD), as corrected by the AlphaSim program with *p* < 0.001 at the voxel-level (cluster-level *p* < 0.05 for a two-tailed test, and a cluster size of >13 voxels). DC values within the regions showing significant differences on analysis of covariance were then extracted as masks and further correlation analysis was performed between DC values and clinical data from the PD groups. Partial correlation analysis was performed in all PD groups after controlling for age, gender, and disease duration; the threshold of significance was set to *p* < 0.05 (using Bonferroni’s correction).

## 3. Results

### 3.1. Demographic and clinical characteristics

There were no significant differences among the three groups in terms of age, gender, and education level (*p* > 0.05). There was also no significant difference in the daily levodopa equivalent dose between the two groups (*p* = 0.32). In addition, there were no significant differences in other clinical variables including disease duration, HY stage, and MMSE scores (*p* > 0.05) between the TD and ARD groups. Significant differences were observed between the two groups in terms of HAMD-17 scores (*p* = 0.02). The ARD group had significantly higher UPDRS and UPDRS-III scores than the TD group (*p* < 0.01 and *p* = 0.01, respectively). Details of the demographic and clinical characteristics of three groups (TD, ARD, and HC) are shown in Table 1.

**Table 1:** Demographic and clinical characteristics of all participants.

Domain	HCs (*n* = 56)	TD group (*n* = 51)	ARD group (*n* = 39)	*F*/*χ*^2^/*Z*	*p*-value
Age (years)	55.00 (50.00, 61.00)	57.00 (52.00, 65.00)	56.36 ± 10.59	3.18	0.20
Sex (M/F)	23/33	30/21	19/20	3.37	0.19
Education (years)	9.00 (6.00, 11.00)	7.45 ± 4.02	6.00 (4.00, 9.00)	3.71	0.16
Duration (years)	–	2.00 (0.50, 3.00)	1.50 (1.00, 3.25)	−0.85	0.39
UPDRS Score	–	18.00 (11.00, 33.50)	27.00 (17.00, 47.50)	−2.93	<0.01
UPDRS-III Score	–	9.00 (6.00, 22.00)	20.00 (10.50, 30.50)	−2.72	0.01
M-TS Score	–	2.00 (1.00, 3.00)	0.50 (0.00, 1.25)	–	–
M-ARS Score	–	0.71 (0.36, 1.86)	1.71 (1.07, 3.29)	–	–
HY stage	–	2.00 (1.00, 2.25)	1.50 (1.50, 2.50)	−1.23	0.22
HAMD-17 Score	–	4.00 (2.50, 7.50)	7.00 (3.00, 17.00)	−2.43	0.02
MMSE Score	–	27.00 (24.00, 28.00)	26.00 (25.00, 28.00)	−0.10	0.92

Data with normal distribution are shown as means ± SD, and not conform to the normal distribution are shown as median (interquartile range). TD, tremor dominan (TD); ARD, akinetic-rigid dominant (ARD); HCs, healthy controls; M, male; F, female; UPDRS, Unified Parkinson’s Disease Rating Scale; UPDRS-III, Unified PD Rating Scale-Part III; M-Ts, mean tremor score; M-ARs, mean akinetic-rigid score; HY, Hoehn and Yahr; HAMD-17, 17-item Hamilton depression scale; MMSE, mini-mental state examination; –, data not available.

### 3.2. Analysis of variance in DC values among the TD, ARD, and HC groups

**Figure 1:**
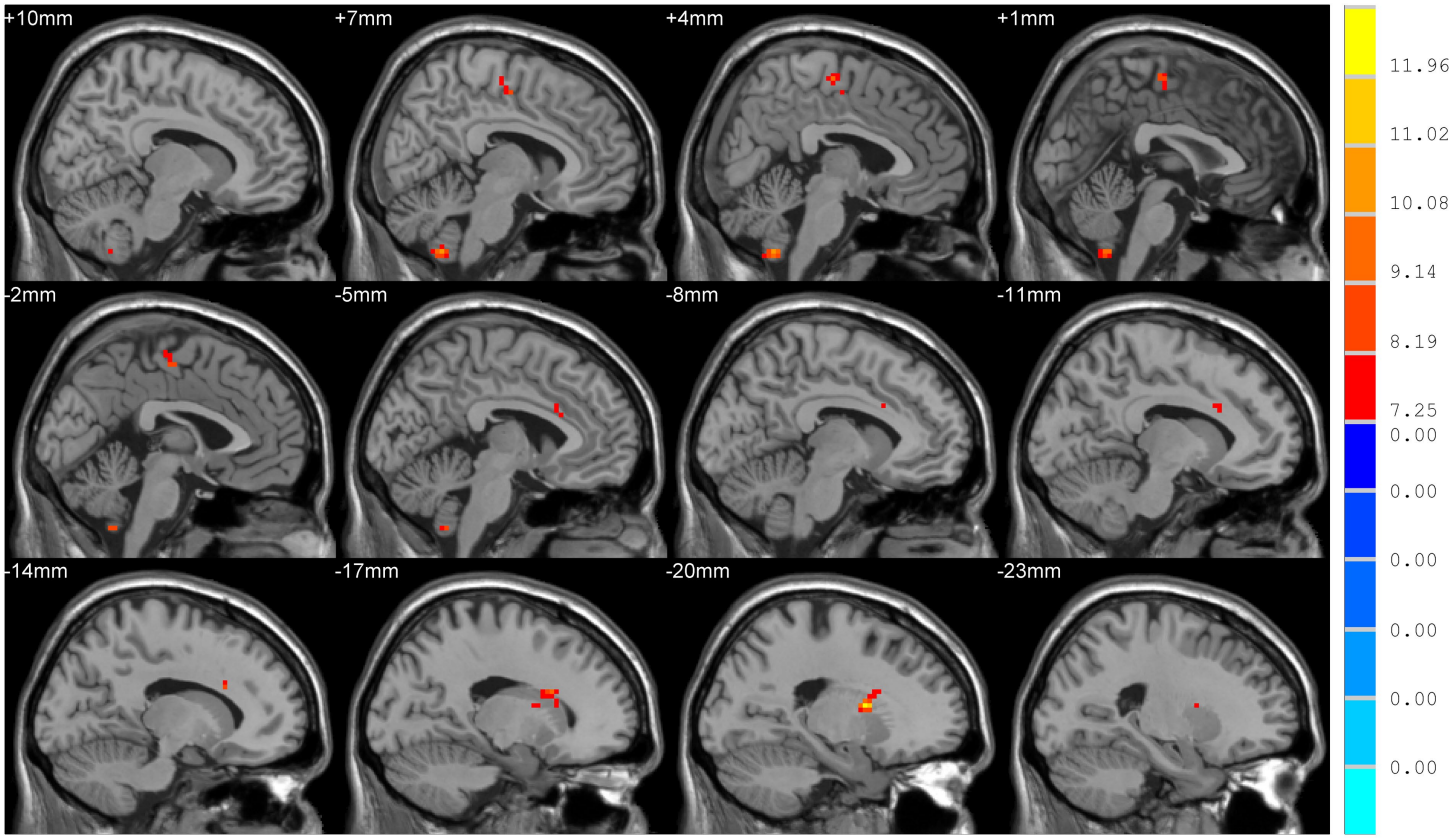
Comparison of degree centrality (DC) among TD, ARD, and HC groups. Significant differences were showed in the following brain regions: bilateral cerebellar hemispheres (Cerebellum_9_R/L), left putamen, left anterior cingulate gyrus, right supplementary motor area, and the left para-central lobule (*p* < 0.05).

The results were visualized using the Statistical Analysis Automated Anatomy Atlas 2 template. Analysis of covariance revealed significant differences among the three groups in terms of DC values of bilateral cerebellar hemispheres (Cerebellum_9_R/L), the left putamen (Putamen_L) extending to the left anterior cingulate gyrus (Cingulum_Ant_L), and the right supplementary motor area (SMA) extending to the left para-central lobule (Para-central_Lobule_L) (Voxel level *p* < 0.001, cluster sizes >13, AlphaSim corrected *p* < 0.05; Figure 1).

### 3.3. Results of the two-sample *t*-test for DC values between two groups (TD vs. HC, ARD vs. HC, and TD vs. ARD groups)

**Figure 2:**
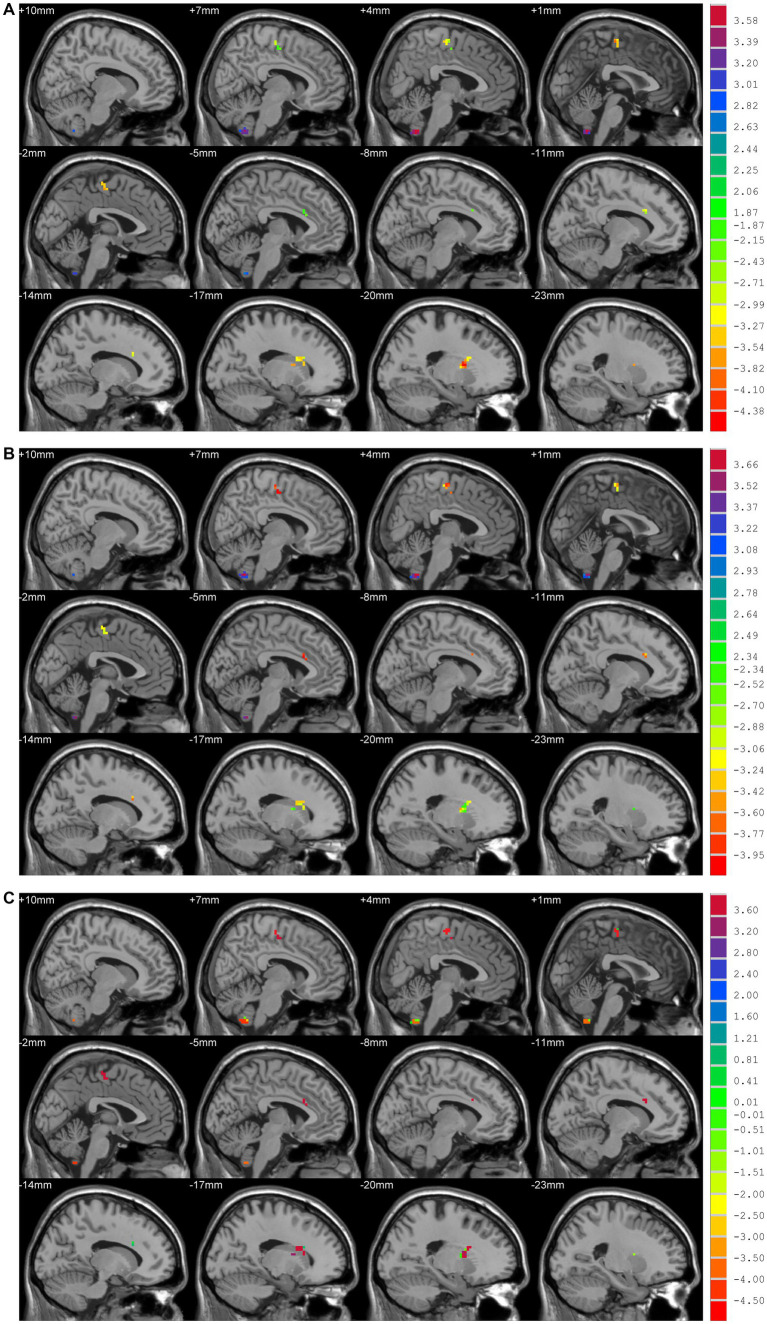
**(A)** TD vs. HCs; DC values had decreased in the Putamen_L and Paracentral_Lobule_L and increased in the Cerebellum_9_R/L. **(B)** ARD vs. HCs; DC values had decreased in the Cingulum_Ant_L and Supp_Motor_Area_R, and increased in the Cerebellum_9_R/L. **(C)** TD vs. ARD groups; DC values had decreased in the Cerebellum_9_R/L, and increased in the Cingulum_Ant_L and Supp_Motor_Area_R.

Compared to HCs, patients in the TD group showed increased DC values in bilateral cerebellar hemispheres and decreased DC values in the left putamen and left para-central lobule. In contrast with HCs, patients in the ARD group showed higher DC values in bilateral cerebellar hemispheres, and lower DC values in the left anterior cingulate gyrus and right SMA. Compared to the ARD group, the TD group showed increased DC values in the left anterior cingulate gyrus and right SMA, and reduced DC values in bilateral cerebellar hemispheres (Voxel level *p* < 0.001, cluster sizes >13, AlphaSim corrected *p* < 0.05; Figure 2 and Table 2).

**Table 2:** Brain regions showing significant DC differences between paired groups from the TD, ARD, and HC groups.

Groups	Brain region (AAL template)	Cluster size	Peak MNI coordinates (*xyz*)		*t*-value	
TD vs. HC						
TD > HC	Cerebellum_9_R/L	26	3	−57	−54	3.7707
TD < HC	Putamen_L	31	−21	3	12	−4.6595
	Paracentral_Lobule_L	24	0	−21	60	−3.8279
ARD vs. HC						
ARD > HC	Cerebellum_9_R/L	26	3	−57	−54	3.8104
ARD < HC	Cingulum_Ant_L	32	−6	18	24	−3.9940
	Supp_Motor_Area_R	24	6	−12	51	−4.1316
TD vs. ARD						
TD > ARD	Cingulum_Ant_L	21	−6	18	24	3.5901
	Supp_Motor_Area_R	18	6	−15	54	3.4045
TD < ARD	Cerebellum_9_R/L	23	−6	−54	−54	−3.612

*x*, *y*, and *z* axes (mm) as proposed by the Montreal Neurological Institute and AlphaSim rectified results. The number of voxels was no less than 13 with a combined threshold of *p* < 0.001, equivalent to *p* < 0.05 after corrected for multiple comparisons.

### 3.4. Correlation analysis

In the TD group, a positive correlation was found between DC values in the right SMA (*x*, *y*, *z* = 3, −18, 60) and the HAMD scale scores (*r* = 0.362, *p* = 0.012). However, the significance did not withstand Bonferroni’s correction. In other clusters of the TD and ARD groups, the correlation between DC values and clinical variables (UPDRS scores, UPDRS-III scores, HY stage, HAMD-17 scores, MMSE scores, TD scores, and AR scores) was not statistically significant. The results were adjusted by Bonferroni’s correction (0.05/7). Detailed correlation data of the two groups are presented in Table 3.

**Table 3:** Detailed correlation data of two groups (TD and ARD).

Domain	TD group (*n* = 51) *r*, *p*-value			ARD group (*n* = 39) *r*, *p*-value		
	Cerebellum_9_R/L	Putamen_L Cingulum_Ant_L	SMA_R Para-central_Lobule_L	Cerebellum_9_R/L	Putamen_L Cingulum_Ant_L	SMA_R Para-central_Lobule_L
UPDRS score	*r* = 0.111, *p* = 0.453	*r* = −0.240, *p* = 0.100	*r* = 0.217, *p* = 0.138	*r* = −0.044, *p* = 0.798	*r* = −0.048, *p* = 0.781	*r* = −0.106, *p* = 0.537
UPDRS-III score	*r* = 0.110, *p* = 0.456	*r* = −0.279, *p* = 0.055	*r* = 0.195, *p* = 0.184	*r* = −0.056, *p* = 0.744	*r* = −0.035, *p* = 0.841	*r* = −0.075, *p* = 0.663
HY stage	*r* = 0.075, *p* = 0.614	*r* = −0.015, *p* = 0.919	*r* = 0.147, *p* = 0.320	*r* = −0.014, *p* = 0.937	*r* = −0.258, *p* = 0.129	*r* = 0.081, *p* = 0.638
MMSE score	*r* = −0.057, *p* = 0.700	*r* = 0.069, *p* = 0.642	*r* = −0.129, *p* = 0.383	*r* = −0.005, *p* = 0.978	*r* = −0.041, *p* = 0.814	*r* = 0.036, *p* = 0.836
HAMD-17	*r* = −0.037, *p* = 0.802	*r* = −0.013, *p* = 0.929	*r* = 0.362, *p* = 0.012	*r* = −0.071, *p* = 0.682	*r* = 0.000, *p* = 0.999	*r* = 0.254, *p* = 0.135
TS	*r* = 0.051, *p* = 0.732	*r* = −0.142, *p* = 0.336	*r* = 0.190, *p* = 0.195	*r* = −0.085, *p* = 0.622	*r* = 0.166, *p* = 0.335	*r* = −0.116, *p* = 0.501
ARS	*r* = 0.113, *p* = 0.443	*r* = −0.259, *p* = 0.075	*r* = 0.253, *p* = 0.083	*r* = 0.032, *p* = 0.854	*r* = 0.070, *p* = 0.685	*r* = −0.181, *p* = 0.292

The partial correlation coefficient and statistical value of *p* were shown above. TD, tremor dominan (TD); ARD, akinetic-rigid dominant (ARD); UPDRS, Unified Parkinson’s disease rating scale; UPDRS-III, Unified PD rating scale-part III; HY, Hoehn and Yahr; MMSE, mini-mental state examination; HAMD-17, 17-item Hamilton depression scale; Ts, tremor score; ARs, akinetic-rigid score.

## 4. Discussion

The findings from this study showed the presence of aberrant intrinsic dysconnectivity among the different motor subtypes of PD from a new perspective of topological properties. Compared to the ARD group, the TD group showed higher DC values in the left anterior cingulate gyrus and right SMA, and lower DC values in bilateral cerebellar hemispheres. To our knowledge, this is the first study to explore alterations in whole-brain synchronization between different subtypes of PD using DC.

The most important finding of this study was the observation of increased DC values in bilateral cerebellar hemispheres of the TD and ARD groups compared to those of HCs. This change was more apparent in the ARD than in the TD group. The role of the cerebellum in PD has recently gained more attention; it is anatomically and functionally interconnected with much of the cortical mantle and basal ganglia, and influences motor planning and execution in addition to a range of higher-order cognitive and emotional functions (O’Callaghan et al., 2016). In the context of the hypoactive cortico-striatal motor circuit, greater activation of the cortico-cerebellar motor circuit is thought to exert a compensatory effect that circumvents the dysfunctional basal ganglia and normalizes motor behavior (Wu and Hallett, 2013). Notably, several studies have found hyperactivity in the primary motor cortex (Sabatini et al., 2000) and cerebellum (Wu and Hallett, 2005) across a variety of motor tasks. Our findings provide additional evidence that the cerebellum demonstrates DC abnormalities in PD (compared to HCs) regardless of motor subtype; however, the abnormalities are more pronounced in the ARD than in the TD group. In comparison with the TD group, the ARD group showed increased DC values in bilateral cerebellar hemispheres. In this context, hyperactivity of the primary motor cortex and cerebellum is also related to specific characteristics of the disease, such as rigidity (Pierantozzi et al., 2001); this may be associated with more obvious abnormal cerebellar DC values in ARD than in TD. As the cerebellum is associated with sensorimotor (Middleton and Strick, 2001), cognitive, and emotional processing (Kansal et al., 2017), changes in its structure and function are associated with gait and motor disorders and some non-motor symptoms in patients with PD. ARD patients show more apparent rigidity and non-motor symptoms than those with TD; this was also verified by the more obvious abnormalities in DC values in the cerebella of the ARD group than in the TD group.

The anterior cingulate gyrus, a key node in the limbic system, plays an important role in emotional regulation and cognition. Compared to the TD and HC groups, the ARD group in our study demonstrated lower DC values in the anterior cingulate gyrus of the left cerebral hemisphere; this indicated that the key nodes involved in emotional regulation were impaired in the ARD group. A significant difference was observed between the TD and ARD groups in terms of HAMD scores (*p* = 0.02). The finding of decreased overall synchronization of the anterior cingulate gyrus in our study was in agreement with the findings from a previous study (Spica et al., 2013); this may explain the higher incidence of depression in ARD patients. As the cingulate cortex is also a key node of the default mode network (DMN) (Laird et al., 2009), the difference suggests inconsistencies in changes to whole brain synchronization in certain key brain regions of the DMN in the two subtypes. Brain activity in the DMN is task negative; that is, there is activation in the resting state and inactivation in the task-related state. Higher overall synchronization of the DMN in patients with PD worsens self-reference ability; this also increases the likelihood of maintaining the default mode state, lessening control on the interaction between brain regions. Decreased FC in the DMN is closely related to cognitive decline (Lucas-Jimenez et al., 2016). Although there was a significant difference in DC values of the anterior cingulate gyrus between the two exercise phenotypes, there was no significant difference in the MMSE score. This may be attributed to the small sample size of this group and the non-significantly shorter duration of disease in patients with TD and ARD PD. Moreover, this could be the ACC is mainly involved (cognitively speaking) in goal-directed behavior and attention allocation, yet the MMSE does not tap on these functions; therefore we will use a more suitable neuropsychological test instead in a future study to investigate how the DC differences in the ACC between the groups reflects on behavior.

This study also found lower DC values in the right SMA of the ARD group than in that of the TD and HC groups. The SMA comprises two different areas (the pre- SMA and SMA) (Lehericy et al., 2004) and is involved in a range of functions including movement, spatiotemporal processing, music and language processing, and working memory (Cona and Semenza, 2017). Recent studies have shown that it plays an irreplaceable role in the integration of affective, behavioral, and cognitive functions (Chung et al., 2005; Nachev et al., 2008). The SMA and striatum are part of a closed-loop motor circuit that is involved in the preparation and self-renewal of future action plans (Forstmann et al., 2008). Some symptoms, such as akinesia and bradykinesia, may be associated with an abnormal SMA (Eggers et al., 2015). A recent study suggested that abnormal motor circuits can also lead to cognitive impairment in patients with PD (Schonberger et al., 2015). Therefore, reduced global synchronization of the SMA may be the key cause for the more rapid motor decline and early onset and development of cognitive impairment in ARD patients. The SMA is critical for planning and initiation of movements; increased activity in this area therefore strongly correlates with the clinical improvement of akinesia (Hou et al., 2018). These findings may reinforce those from previous studies and support the existing notion that the TD phenotype has a better prognosis and demonstrates slower disease progression than the ARD phenotype (owing to compensation by the SMA in the former). We believe that the clinical symptoms in ARD patients, that include a rapid decline in the amount of exercise, may be related to the reduction of DC values in the SMA. The ARD subtype showed decreased connections in the right SMA compared to the TD subtype; this was associated with typical symptoms of akinesia and bradykinesia in the former.

Compared to the HCs, the TD group showed decreased DC values in the left putamen and para-central lobule. Unsurprisingly, abnormal DC values were found in the putamen; as described in previous studies, the putamen has an abundance of dopaminergic neurons and their dysfunction has been consistently demonstrated in PD. In this context, the abnormalities in the putamen are considerably more frequent and severe than those of the caudate nucleus (Sasannezhad et al., 2017). In view of the results from the present and previous studies, it is reasonable to conclude that abnormal DC values in the putamen form an important node in the cortico-striatal-thalamo-cortical circuit disorder of TD. Decreased DC values were found in the left para-central lobule in the TD group. In this context, the para-central lobule is a part of the primary somatic sensory area; it controls skeletal muscle movement in the contralateral limb (including movement and sensation in the contralateral calves and feet). Cases with impaired para-central lobule function demonstrate contralateral lower extremity weakness and dysfunction in urination and defecation; certain non-motor symptoms in TD patients may also be explained by impaired para-central lobule function.

There are some limitations to this study. First, although the patients in our study had stopped anti-PD drugs for 12 h prior to MR examination and clinical neuropsychological evaluation, we were unable to estimate the residual effects of the drugs; this may have skewed our results. Second, this study had a cross-sectional design; this precluded characterization of the dynamic profiles of DC in different motor types of PD. Further longitudinal studies are therefore needed to explore the dynamic characteristics of different subtypes of PD. Third, although the AlphaSim-corrected significance threshold (at *p* < 0.05) combined with the threshold of *p* < 0.001 (at voxel level) and the corresponding cluster sizes employed were acceptable (Chung et al., 2005; Cona and Semenza, 2017), the stringency was limited to a certain extent. Our results may therefore be generalized with caution; further replication studies are needed to validate our findings.

This study demonstrated that DC varied widely at the voxel level in the different subtypes of PD (TD and ARD); this correlated highly with the clinical heterogeneity of different motor subtypes. This study therefore provides new concepts for further study of the neuropathological mechanisms in different subtypes of PD.

## Data availability statement

The raw data supporting the conclusions of this article will be made available by the authors, without undue reservation.

## Author contributions

CS, FZ, CT, JL, LZ, MW, JY, and YL: data collection. CS, QS, TW, and SC: data analysis. CS and QS: manuscript writing. HL: project development and manuscript revision. All authors contributed to the article and approved the submitted version.

## Footnotes

^1^ http://www.fil.ion.ucl.ac.uk/spm/
