# Supplementary material for: Distinct changes in global brain synchronization in different motor subtypes of Parkinson’s disease
Source: Front Neurosci. 2023 Oct 18;17:1170225. doi: 10.3389/fnins.2023.1170225 (PMC10618346; doi:10.3389/fnins.2023.1170225)
Supplement: Supplementary file 2 [file Data_Sheet_1.PDF]

GRF（voxel P=0.01，cluster：P=0.05）

1、TD-ARD-HC：

```
FWHMx = 1.713768 voxels
FWHMy = 1.828103 voxels
FWHMz = 1.778986 voxels
FWHMx = 5.141303 mm
FWHMy = 5.484309 mm
FWHMz = 5.336959 mm
DLH = 0.828329
VOLUME = 70831
RESELS = 5.573465
The voxel Z threshold for voxel p threshold 0.010000 is: 2.575829.
The Minimum cluster size for voxel p threshold 0.010000 and cluster p threshold 0.050000 is: 26.000000 voxels
This report is based on CUI Xu's xjview. (http://www.alivelearn.net/xjview/)
Revised by YAN Chao-Gan and ZHU Wei-Xuan 20091108: suitable for different Cluster Connectivity Criterion: surface connected, edge co
Number of clusters found: 4
```

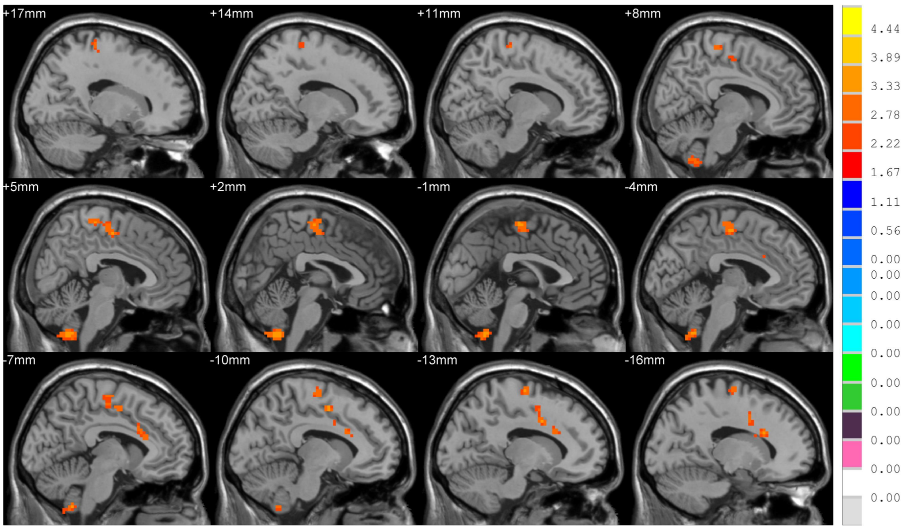

Cluster 1：

Cerebelum_9_R/L (aal)

Number of voxels: 90

Peak MNI coordinate: 3 -57 -54

Peak intensity: 3.8702

Cluster 2：

Putamen_L (aal)

Number of voxels: 88

Peak MNI coordinate: -21 6 12

Peak intensity: 4.0277

Cluster 3：

Left Cingulate Gyrus

Number of voxels: 33

Peak MNI coordinate: -12 3 36

Peak intensity: 3.4799

Cluster 4：

Supp_Motor_Area_R (aal)

Number of voxels: 144

Peak MNI coordinate: 3 -18 60

Peak intensity: 3.5615

2、TD-HC

```
...........................................................
FWHMx = 1.402434 voxels
FWHMy = 1.454699 voxels
FWHMz = 1.373575 voxels
FWHMx = 4.207302 mm
FWHMy = 4.364096 mm
FWHMz = 4.120726 mm
DLH = 1.647480
VOLUME = 70831
RESELS = 2.802257
The voxel Z threshold for voxel p threshold 0.010000 is: 2.575829.
The Minimum cluster size for voxel p threshold 0.010000 and cluster p threshold 0.050000 is: 15.000000 voxels
This report is based on CUI Xu's xjview. (http://www.alivelearn.net/xjview/)
Revised by YAN Chao-Gan and ZHU Wei-Xuan 20091108: suitable for different Cluster Connectivity Criterion: surface connected, edge cor
Number of clusters found: 3
```

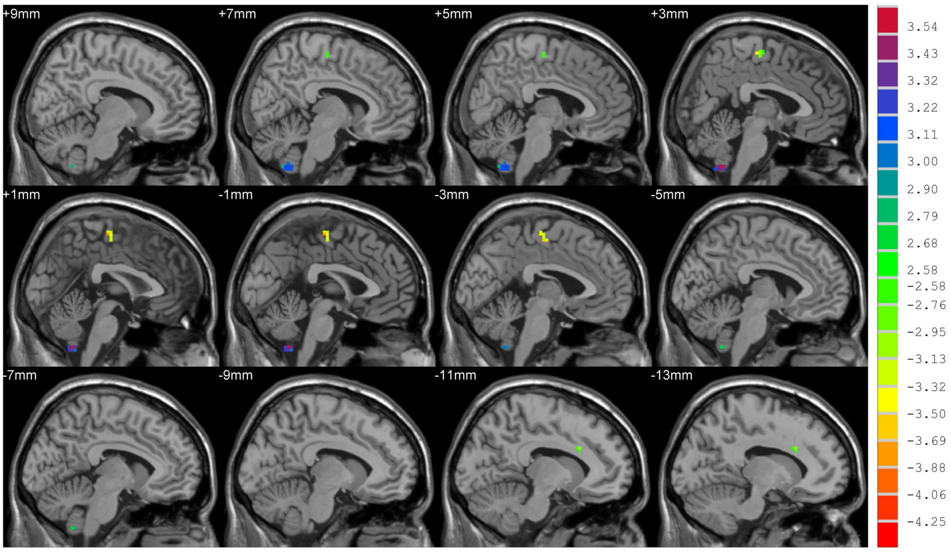

TD>HC:

Cluster 1：

Cerebelum_9_R/L (aal)

Number of voxels: 25

Peak MNI coordinate: 0 -57 -54

Peak intensity: 3.5658

TD<HC:

Cluster 1：

Putamen_L (aal)

Number of voxels: 28

Peak MNI coordinate: -21 6 12

Peak intensity: -4.3539

Cluster 2：

Paracentral_Lobule_L (aal)

Number of voxels: 20

Peak MNI coordinate: 0 -21 60

Peak intensity: -3.6949

3、ARD-HC:

```
........................................................
FWHMx = 1.417843 voxels
FWHMy = 1.294608 voxels
FWHMz = 1.267786 voxels
FWHMx = 4.253529 mm
FWHMy = 3.883823 mm
FWHMz = 3.803357 mm
DLH = 1.983883
VOLUME = 70831
RESELS = 2.327085
The voxel Z threshold for voxel p threshold 0.010000 is: 2.575829.
The Minimum cluster size for voxel p threshold 0.010000 and cluster p threshold 0.050000 is: 13.000000 voxels
This report is based on CUI Xu's xjview. (http://www.alivelearn.net/xjview/)
Revised by YAN Chao-Gan and ZHU Wei-Xuan 20091108: suitable for different Cluster Connectivity Criterion: surface connected, edge co
Number of clusters found: 3
-----------------------
```

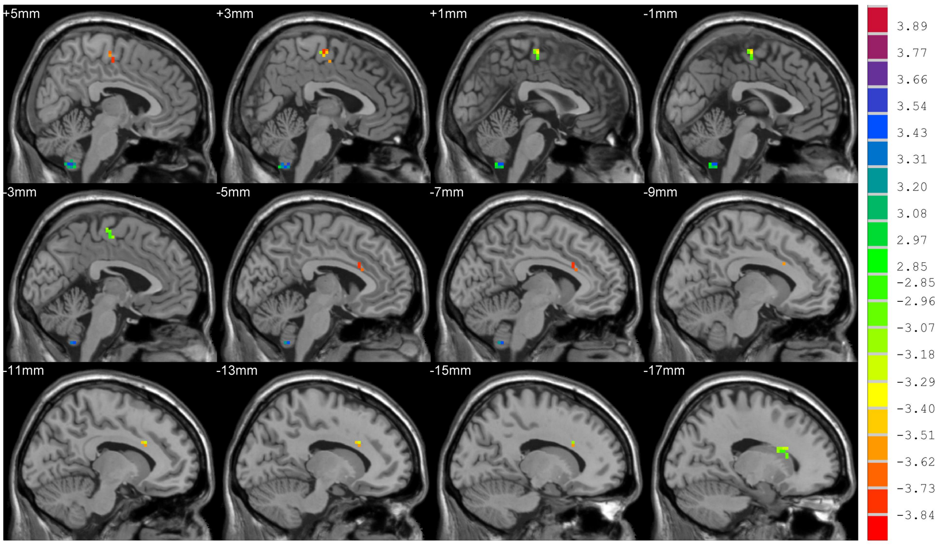

ARD>HC:

Cluster 1：

Cerebelum_9_R/L (aal)

Number of voxels: 25

Peak MNI coordinate: 6 -57 -54

Peak intensity: 3.6262

ARD<HC:

Cluster 1：

Cingulum_Ant_L (aal)

Number of voxels: 21

Peak MNI coordinate: -6 18 24

Peak intensity: -3.8269

Cluster 2：

Supp_Motor_Area_R (aal)

Number of voxels: 23

Peak MNI coordinate: 6 -15 51

Peak intensity: -3.811

4、TD-ARD

There are no meaningful results.
